# Different Transcriptomic Responses After Moderate and Severe Heat Shock in *Trypanosoma cruzi* Epimastigotes

**DOI:** 10.1111/jeu.70125

**Published:** 2026-09-09

**Authors:** Thayane Bottaro, Luciana L. Penha, Rosane Silva, Turán P. Ürményi

**Affiliations:** ^1^ Instituto de Biofísica Carlos Chagas Filho Universidade Federal do Rio de Janeiro Rio de Janeiro Brazil

**Keywords:** gene expression, heat shock response, transcriptome profile

## Abstract

*Trypanosoma cruzi* undergoes heat stress during its life cycle. In this study, we performed a transcriptomic analysis of the heat shock response in epimastigotes from different *T. cruzi* strains. In both clone CL Brener and the Y strain, very few to no statistically significant variations were observed in the transcriptome at 37°C, whereas differentially expressed genes were observed at 40°C, with 1018 upregulated and 734 downregulated transcripts in CL Brener and 1426 upregulated and 1223 downregulated transcripts in the Y strain. Upregulated transcripts were enriched in catabolic pathways, whereas downregulated transcripts were enriched in catabolic and anabolic pathways. In addition, the expression pattern of different members of several heat shock protein gene families varied. The expression patterns of members of surface protein gene families, such as trans‐sialidases, GP63, mucins, and mucin‐associated proteins, also varied, although the physiological significance of these patterns remains unclear. We conclude that, in *T. cruzi* epimastigotes, transcriptome modulation at 37°C was less pronounced than expected for an 8°C increase in temperature, whereas at 40°C, there was functional enrichment in both induced and repressed transcripts, characterizing a classical response to severe heat shock.

## 1 Introduction

*Trypanosoma cruzi* is a flagellated protozoan parasite and a member of the order Kinetoplastida. It is the causative agent of Chagas disease, with millions of people infected worldwide, and no effective treatment or vaccine is available (Coura and Vinas 2010). The parasite has a complex life cycle, infecting an insect vector and a mammalian host and undergoing differentiation through several developmental stages (De Souza 2002). The epimastigote form in the insect differentiates into metacyclic trypomastigotes, the infective forms in the mammal. Once inside a mammalian host cell, they differentiate into amastigotes, proliferate, differentiate into bloodstream trypomastigotes, and are released into the bloodstream. The released trypomastigotes invade another host cell, maintaining the infection, or are ingested by the insect vector in a blood meal. Once inside the insect, the cells differentiate into epimastigotes, which proliferate.

During its life cycle, the parasite undergoes several kinds of stress against which a range of cellular responses are induced. The cellular stress response is transient, specific to the damaged macromolecule, and results in stress tolerance, whereas the cellular homeostasis response is specific to the environmental stressor and leads to adaptation (Kultz 2005). *T. cruzi* 's heat and oxidative stress responses are examples of the former, and nutritional and osmotic stress responses of the latter. Heat stress induces molecular chaperones and proteases in the parasite, whereas oxidative stress induces small redox molecules and antioxidant enzymes (Ürményi et al. 2012; Jimenez 2014). The nutritional stress response consists of the formation of P‐bodies/stress granules and induction of autophagy‐related genes, and the osmotic stress response involves the efflux of ions and osmolytes and the release of water by a contractile vacuole and the acidocalcisomes (Ürményi et al. 2012; Jimenez 2014). In addition to metacyclic trypomastigotes, which are subjected to heat stress during the transition from the insect to the mammalian host, epimastigotes in the insect host are also subjected to changes in ambient temperature and thus are good models for studying the heat stress response in this organism.

In trypanosomatids, gene expression and regulation show several peculiar mechanisms. Protein‐coding genes are transcribed by RNA polymerase II into polycistronic transcripts that mostly originate from strand‐switch regions in the chromosome, although some transcription may initiate within gene clusters (Herreros‐Cabello et al. 2020). These transcripts are processed to individual mRNAs by polyadenylation and trans‐splicing, in which a 39‐nucleotide sequence (called mini‐exon or spliced leader) is added to the 5′ end of each mRNA. No RNA polymerase II promoter has been identified in these organisms, and gene regulation occurs at the post‐transcriptional level (primarily through mRNA stability and translation; Herreros‐Cabello et al. 2020).

The heat shock response is usually triggered by a temperature increase of only a few degrees, which is sufficient to cause protein unfolding, nonspecific aggregation, and a general loss of protein homeostasis (Richter et al. 2010). In addition to a general decrease in translation and changes in membrane morphology and fluidity, this loss of homeostasis leads, in eukaryotes, to cytoskeleton disorganization, fragmentation of the Golgi apparatus and the endoplasmic reticulum, and reduced numbers of mitochondria and lysosomes. In an attempt to counter these effects, several classes of protein genes are induced: (a) molecular chaperones to refold denatured proteins; (b) components of the proteolytic system to degrade irreversibly aggregated proteins; (c) RNA‐ and DNA‐modifying enzymes for DNA repair and correct RNA processing defects; (d) metabolic enzymes to reorganize the energy supply; (e) regulatory proteins of gene expression and signaling pathways; (f) cytoskeleton‐related proteins; and (g) transport, detoxifying, and membrane‐modulating proteins to restore membrane function (Richter et al. 2010).

Several *T. cruzi* HSP genes and their expression patterns have been characterized by our group and others (Folgueira and Requena 2007; Urmenyi et al. 2014). Increased levels of HSP60, HSP70, HSP85, and HSP100 are observed in epimastigotes subjected to a heat shock from 37°C to 43°C, with the cells becoming round and showing reduced motility at 40°C and above. In contrast to other eukaryotes, HSPs are present in non‐heat‐shocked cells, and the typical inhibition of translation is seen only at 40°C and above. The number of HSP genes in the *T. cruzi* genome varies, from one (HSP100) to 6–9 (HSP85 and HSP70) to more than 50 (HSP40). HSP100 mRNA levels increase at 37°C and 40°C, whereas those of HSP70 increase at 37°C but not at 40°C. The increased HSP70 mRNA levels result, at least in part, from increased stability conferred by elements in the 5′ and 3′ UTRs (Rodrigues et al. 2010). Conflicting reports have described HSP60 mRNA as either increased or unchanged after heat shock, whereas HSP10 mRNA levels are reported to be unchanged. Since HSP60 and HSP10 comprise a single functional unit, they are speculated to be coordinately regulated. Finally, sHSP16 mRNA levels increase slightly at elevated temperatures.

Few large‐scale studies of the heat shock response in *T. cruzi* have been reported. Proteomic analysis of epimastigotes subjected to increased temperatures identified 19 proteins with increased levels and 4 with decreased levels belonging to the following functional categories: metabolism, cell defense, protein fate, protein synthesis, cellular transport, and cell cycle (Perez‐Morales et al. 2012). In the transcriptomic analysis of metacyclic trypomastigotes, a slight increase in temperature from 27°C to 28°C results in upregulation of GP63 and downregulation of genes involved in lipid and carbohydrate metabolism, oxidative stress, proteolysis, phosphorylation, and translation (Cruz‐Saavedra et al. 2020).

*T. cruzi* exhibits extensive interstrain genotypic and phenotypic heterogeneity (Reis‐Cunha et al. 2018; Herreros‐Cabello et al. 2020), which includes varying responses to different kinds of stress. Therefore, in this study, we investigated the transcriptome profiles of *T. cruzi* epimastigotes from two strains subjected to elevated temperatures to better understand the parasite's basic physiological response to heat shock and to evaluate potential strain‐dependent variability in this response. The temperatures of 29°C, 37°C, and 40°C were selected based on the parasite's life cycle and physiological conditions. The temperature of 29°C corresponds to the standard culture condition for epimastigotes and reflects the typical ambient temperature of the invertebrate vector. The increase to 37°C simulates a moderate heat‐stress condition, close to mammalian body temperature, allowing investigation of how the parasite responds to physiological variations it might encounter in these hosts. Finally, 40°C represents a severe heat shock, mostly above the physiological range, used to trigger classical heat shock protein responses and significant transcriptomic changes, enabling the identification of genes and pathways involved in adaptation to extreme stress. Additionally, 40°C is a common temperature in tropical countries and falls within the range tolerated by the triatomine insect vector. This temperature selection provides a controlled gradient that distinguishes basal, moderate, and severe responses to thermal increase.

## 2 Materials and Methods

### 2.1 Parasites and Culture Conditions

Epimastigote cells of *T. cruzi* clone CL Brener and Y strain (DTUs VI and II, respectively) (Zingales et al. 2009) were maintained at 29°C in liver infusion tryptose medium supplemented with 10% bovine calf serum, 25 μg/mL hemin, and 100 μg/mL ampicillin. Cells from early‐exponential‐phase cultures were incubated at different temperatures for 3 h, and their viability after treatment was confirmed using the LIVE/DEAD Viability/Cytotoxicity Kit (Thermo Fisher Scientific).

### 2.2 RNA Extraction, Library Preparation, and Sequencing

Total RNA was extracted from epimastigotes using TRIzol reagent (Thermo Fisher Scientific) according to the manufacturer's instructions. Experiments were performed in three biological replicates. RNA samples were enriched for poly(A) + RNA using the MicroPoly(A) Purist kit (Thermo Fisher Scientific) according to the manufacturer's instructions. The integrity of the RNA samples was evaluated using a 2100 Bioanalyzer system (Agilent), with corresponding RNA integrity numbers > 7. RNA‐seq libraries were constructed using the Ion Total RNA‐seq v2 kit (Thermo Fisher Scientific). Briefly, poly(A) + RNA was fragmented with RNase III, ligated to adaptors, and converted to cDNA. The size distribution of the cDNA fragments was evaluated using a 2100 Bioanalyzer system (Agilent). Clonal amplification of the RNA‐seq library was performed by emulsion PCR using the Ion OneTouch 200 Template kit in an Ion OneTouch 2 System (Thermo Fisher Scientific). Libraries were sequenced with the Ion PGM Sequencing 200 v2 kit in an Ion Torrent Personal Genome Machine (Thermo Fisher Scientific). Raw data were submitted to the GEO database under accession number GSE195949.

### 2.3 Quality Control and Read Mapping

Adapter, spliced leader, poly(A) sequences, and reads with length below 25 bp or quality scores below 20 were removed, and reads were trimmed using the Torrent Suite software v5.0 (Thermo Fisher Scientific) and CLC Genomics Workbench software v8.5 (Qiagen). Trimmed reads were mapped to the *T. cruzi* CL Brener Esmeraldo‐like reference genome v46 (www.tritrypdb.org) using the CLC Genomics Workbench software v8.5 with the following parameters: mismatch cost 2, insertion cost 3, deletion cost 3, minimum overlap length fraction 0.5, and minimum similarity fraction 0.8. The total number of mapped reads for each transcript was counted and normalized to obtain reads per million (RPM) and reads per kilobase per million (RPKM).

### 2.4 Differential Gene Expression, Gene Ontology Enrichment, and Pathway Enrichment Analyses

Differential expression analyses were performed using the RNA‐seq and expression analysis modules of CLC Genomics Workbench, as well as the edgeR and EnhancedVolcano Bioconductor R packages. The empirical analysis of differential gene expression (EDGE) tool implements the Exact Test with a negative binomial distribution for two‐group comparisons (Robinson and Smyth 2008), using a total‐count filter cutoff of 2 for both common dispersion and estimated tagwise dispersions. Raw counts were used. All *p*‐values obtained were adjusted for false discovery rate (FDR) (Benjamini and Hochberg 1995), and differential gene expression values were considered significant with fold‐change > 1.5 and FDR‐adjusted *p*‐value < 0.05. Gene ontology (GO) enrichment and pathway enrichment analyses of up‐ and downregulated genes were performed using the EuPathDB online tool (Aurrecoechea et al. 2017) with Fisher's Exact test, adjusted for FDR, at *p*‐value < 0.05.

## 3 Results

### 3.1 Sequencing and Mapping to the Reference Genome

**FIGURE 1:**
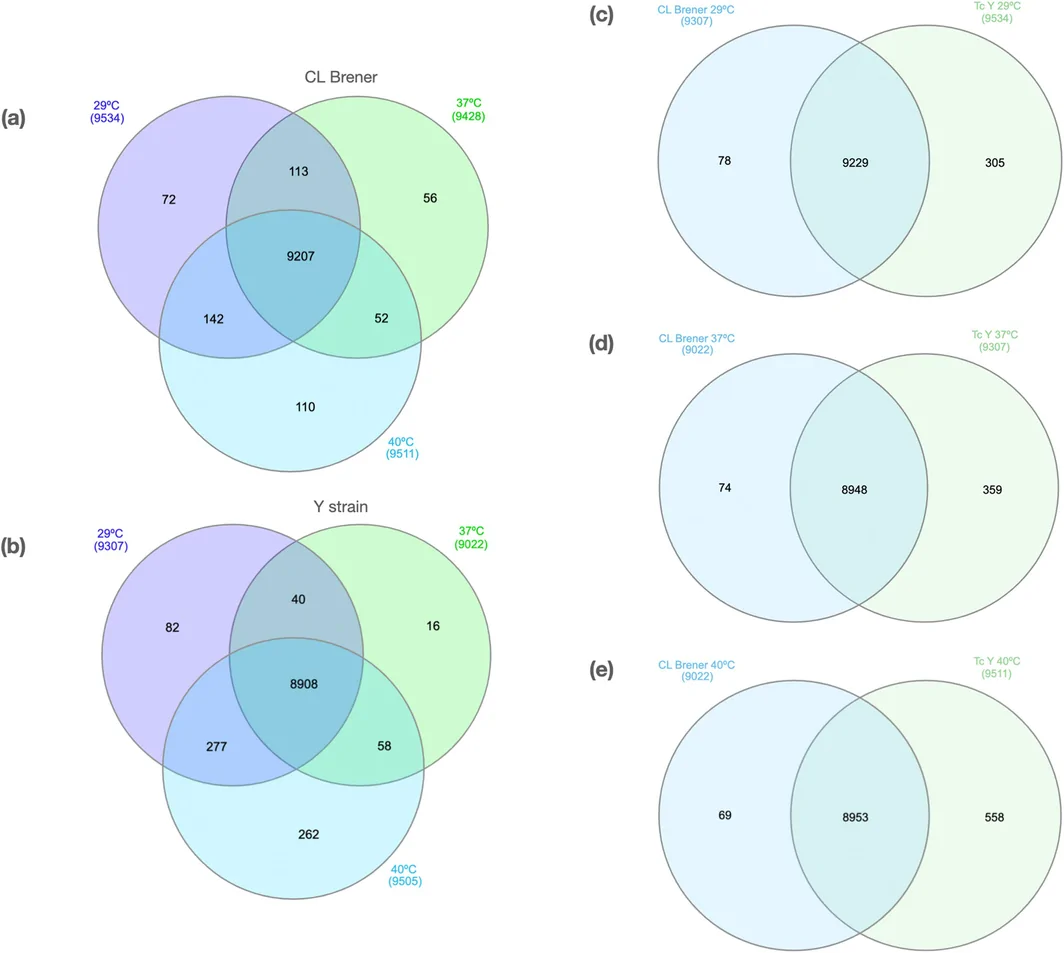
Number of gene transcripts in different temperatures in CL Brener and Y strain epimastigotes. (a) Number of gene transcripts detected at different temperatures in CL Brener. (b) Number of gene transcripts detected at different temperatures in the Y strain. (c) Number of gene transcripts detected at 29°C in CL Brener and the Y strain. (d) Number of gene transcripts detected at 37°C in CL Brener and the Y strain. (e) Number of gene transcripts detected at 40°C in CL Brener and the Y strain. Numbers in parentheses indicate the total number of transcripts with at least two reads per million of total reads detected at that temperature.

A total of 44,451,412 reads were obtained from three replicates (Figure S1) of heat‐treated and control *T. cruzi* epimastigotes of clone CL Brener and the Y strain after quality and size trimming. Average read length ranged from 62.7 to 115.3 bp, and at least 80% of reads were mapped to the reference genome, which therefore can be considered a bona fide genome reference (Table S1). The transcript detection threshold was 5 raw read counts across all samples. Transcripts were detected in CL Brener epimastigotes for most of the 10,831 annotated protein‐coding genes per haploid genome, with 9207 (85%) genes common to all temperatures and 72, 56, and 110 detected only in cells at 29°C, 37°C, and 40°C, respectively (Figure 1a). For the Y strain, transcripts were detected in epimastigotes for 8908 (82%) genes common to all temperatures and 82, 16, and 262 detected only in cells at 29°C, 37°C, and 40°C, respectively (Figure 1b). At 29°C, 9229 transcripts were detected in both cell lineages, with 78 and 305 being detected only in CL Brener and the Y strain, respectively (Figure 1c). At 37°C, 8948 transcripts were detected in both cell lineages, with 74 and 359 being detected only in CL Brener and the Y strain, respectively (Figure 1d). At 40°C, 8953 transcripts were detected in both cell lineages, with 69 and 558 being detected only in CL Brener and the Y strain, respectively (Figure 1e).

### 3.2 Differential Gene Expression

**FIGURE 2:**
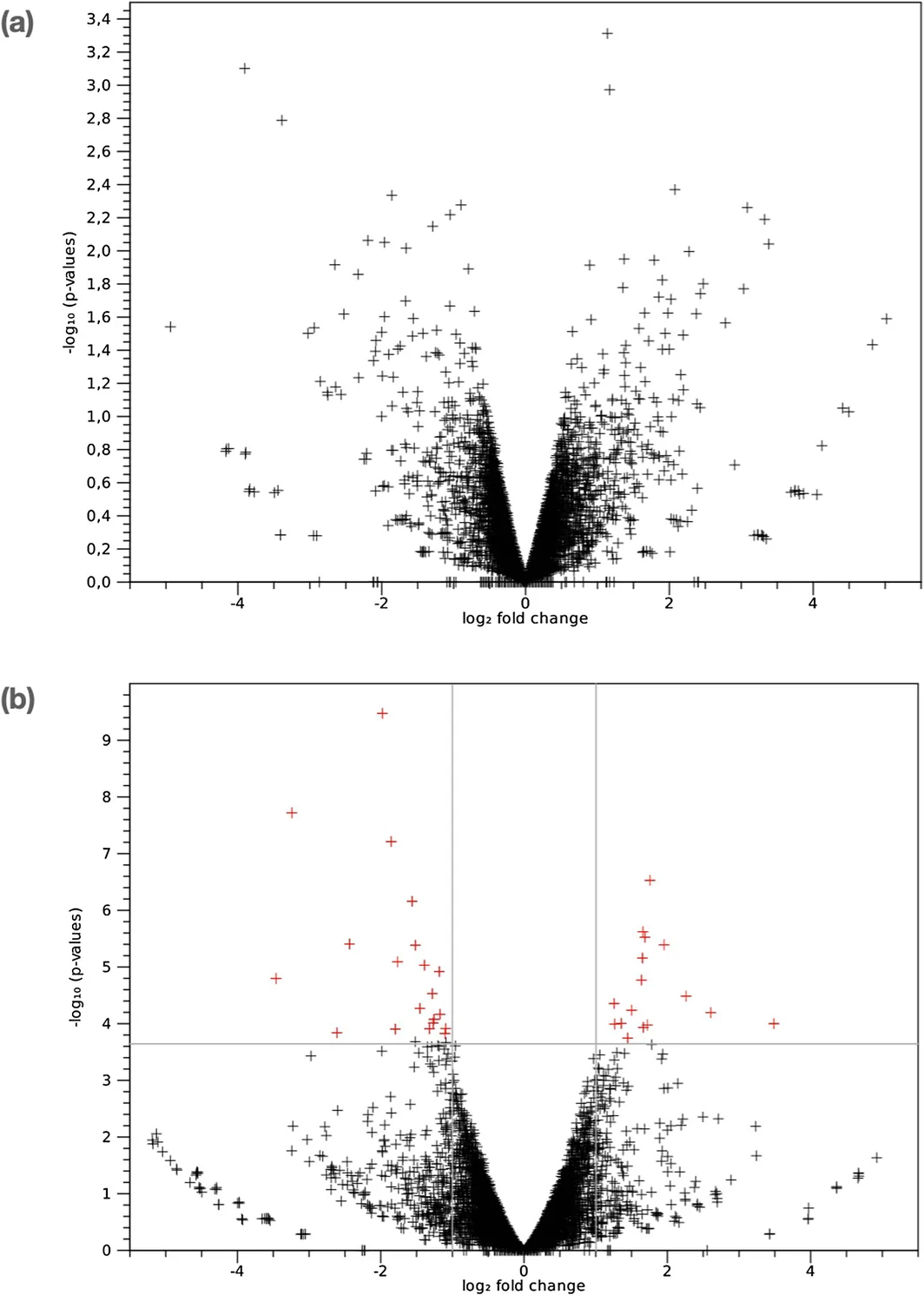
Differential gene expression of epimastigotes incubated at 37°C relative to that at 29°C. Volcano plots show gene expression variation of CL Brener (a) and Y strain (b) epimastigotes relative to statistical significance (EDGE test). Statistical significance is shown as −log_10_ of *p*. Genes with statistically significant variation (FDR‐corrected *p* < = 0.05) and fold‐change > 1.5 or < −1.5 are shown in red.

**FIGURE 3:**
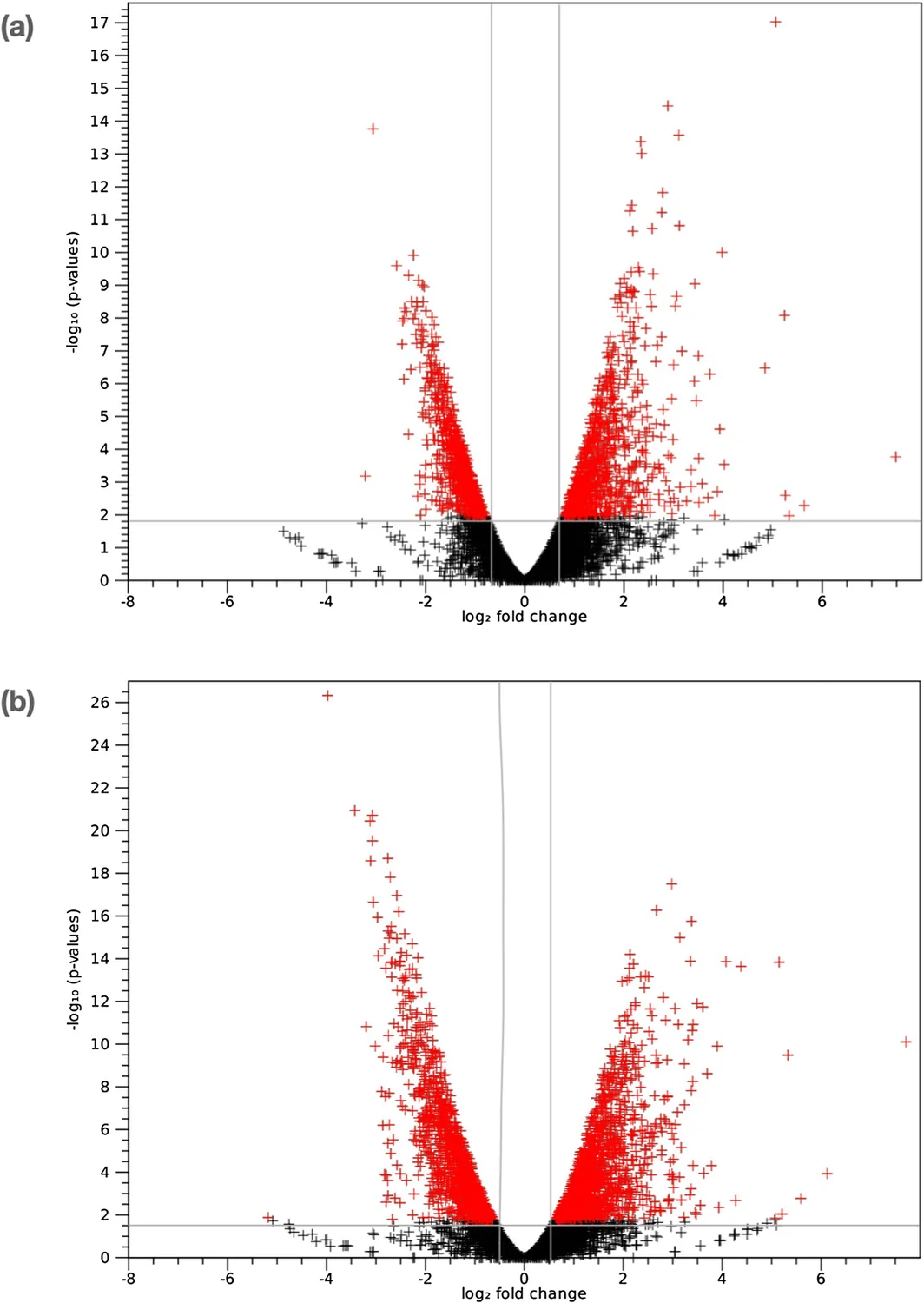
Differential gene expression of epimastigotes incubated at 40°C relative to that at 29°C. Volcano plots show gene expression variation of CL Brener (a) and Y strain (b) epimastigotes relative to statistical significance (EDGE test). Statistical significance is shown as −log_10_ of *p*. Genes with statistically significant variation (FDR‐corrected *p* < = 0.05) and fold‐change > 1.5 or < −1.5 are shown in red.

Differential gene expression profiles of epimastigotes subjected to heat shock were similar between clone CL Brener and the Y strain. Surprisingly, in EDGE analysis, when FDR‐adjusted *p*‐values were used, no statistically significant variation was observed at 37°C in CL Brener (Figure 2a) and only in a few gene transcripts in the Y strain (Figure 2b, red crosses), even though this heat shock corresponds to a temperature shift of 8°C. In contrast, differentially expressed gene transcripts were observed at 40°C (Figure 3, red crosses) in both CL Brener (Figure 3a) and the Y strain (Figure 3b) epimastigotes when compared with 29°C. A total of 1752 differentially expressed genes were observed in CL Brener epimastigotes, with 1018 being upregulated and 734 downregulated. In Y strain epimastigotes, 2649 differentially expressed genes were identified, with 1426 upregulated and 1223 downregulated.

### 3.3 GO and Pathway Enrichment Analyses

**FIGURE 4:**
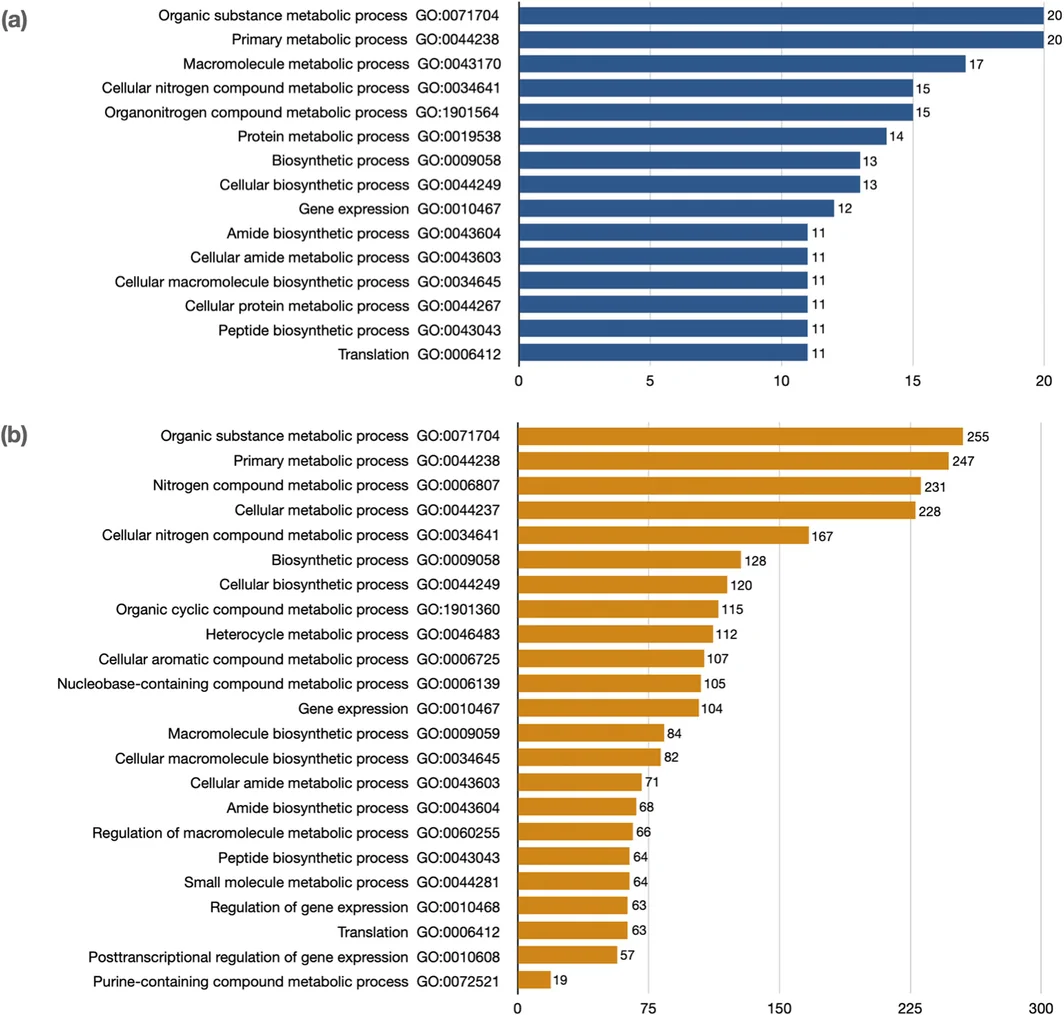
Gene set enrichment analysis of differentially expressed genes in epimastigotes subjected to heat shock. Gene ontology (GO) terms for biological processes enriched in up‐ (a, blue) and downregulated (b, orange) genes in CL Brener epimastigotes incubated at 40°C are shown (*p* < 0.001). The number of enriched genes is shown to the right of each horizontal bar.

Gene ontology and pathway enrichment analyses were performed on the upregulated and downregulated genes of epimastigotes subjected to heat shock. Because clone CL Brener and the Y strain showed highly similar patterns, only the results from clone CL Brener are shown. The 677 upregulated genes at 40°C were mostly enriched in the GO biological processes of organic and nitrogen compound metabolism, macromolecule metabolism, and protein metabolism (Figure 4a, blue). In pathway enrichment analysis, these processes correspond to pathways related to the degradation of steroids, hemoglobin, glyconate, and phosphonate, as well as to sulfonate compound metabolism, such as cysteine biosynthesis. Although the 458 downregulated genes were mostly enriched in the same GO biological processes, they were also enriched in several biosynthetic processes related to macromolecules and nitrogen compounds (Figure 4b, orange). These processes correspond to pathways involved in amino acid degradation, acetyl‐CoA biosynthesis, glycolysis, gluconeogenesis, the pentose phosphate pathway, nucleotide metabolism, the tricarboxylic acid cycle, fatty acid degradation, and the degradation of cholesterol and other steroids. In general, as described for severe heat shock in other eukaryotes, the altered gene expression pattern at 40°C in the parasite reflects macromolecule homeostasis and reorganization of metabolic pathways involved in the synthesis of energy‐rich compounds. In addition, gene transcripts from several lipid metabolism‐related pathways were modulated (Tables S1 and S2), suggesting a homeoviscous adaptation in which membrane composition is altered to preserve proper fluidity.

### 3.4 Heat Shock Protein Gene Expression

The *T. cruzi* genome contains several large gene families with hundreds to thousands of copies, exhibiting some sequence heterogeneity (Herreros‐Cabello et al. 2020). This heterogeneity is sufficient to identify the gene copy from which a transcript originates.

**FIGURE 5:**
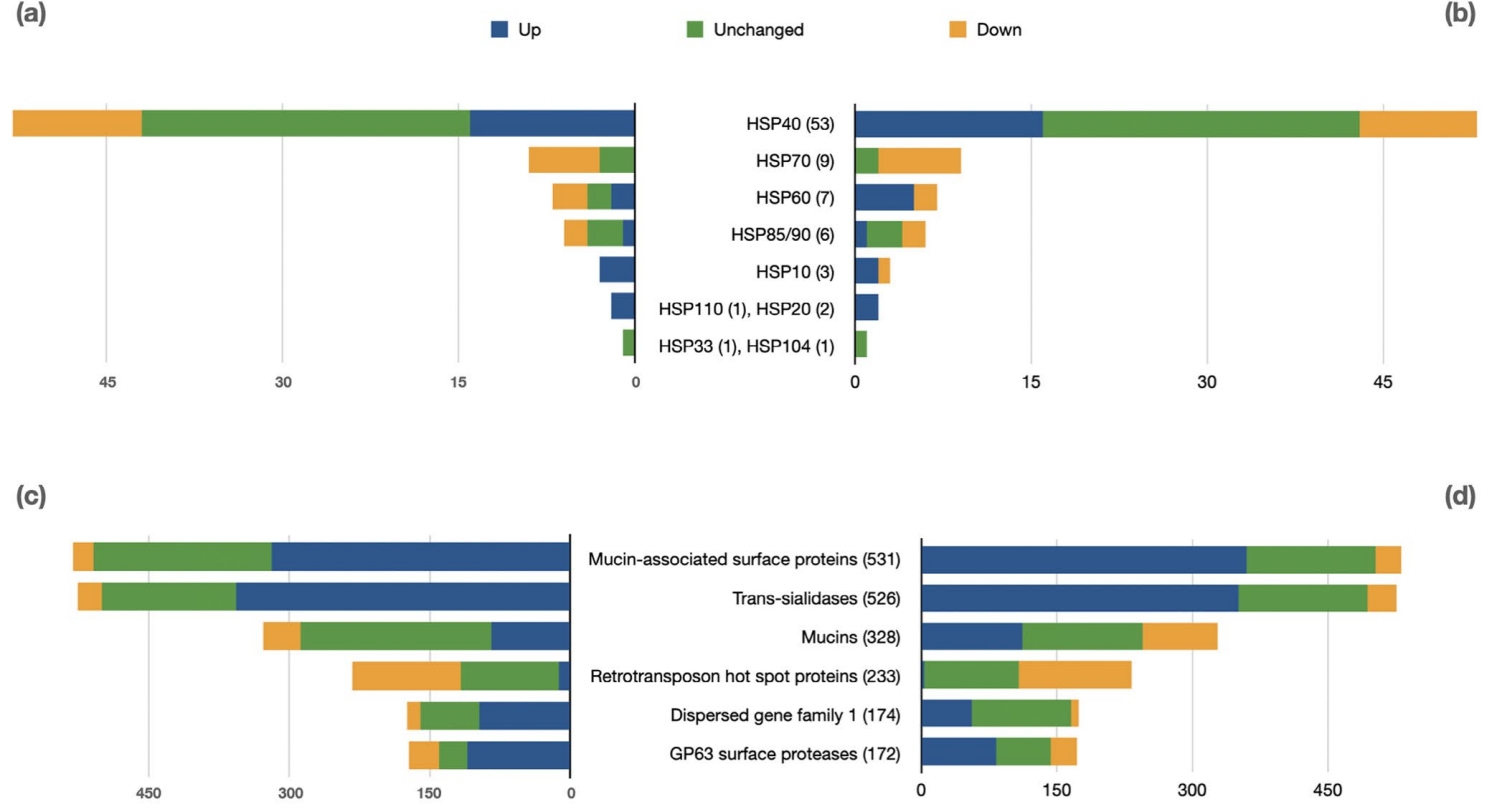
Transcript level variation of HSP and surface protein genes in epimastigotes at 40°C. The bar graph shows the number of HSP (a,b) and surface protein (c,d) gene family members with increased (blue) or decreased (orange) transcript levels in CL Brener (a,c) and Y strain (b,d) epimastigotes incubated at 40°C (FDR‐corrected *p* < = 0.05). The number of genes with unaltered transcript levels is shown in green. Numbers in parentheses indicate the total number of gene family members annotated in the genome from which transcripts were detected.

The *T. cruzi* orthologs of the major heat shock protein (HSP) gene families are organized and named based on sequence similarity and molecular weight (Folgueira and Requena 2007). HSPs are responsible for helping proteins reach their native conformation after synthesis and for maintaining protein homeostasis under heat stress, either by restoring their native conformation or by degrading misfolded proteins. Differential gene expression analysis shows that the expression pattern of HSP gene family members varied when clone CL Brener and Y strain epimastigotes were subjected to heat shock. As expected, transcripts from the HSP110, HSP20, and HSP10 genes were mostly upregulated at 40°C (Figure 5a,b, blue). Also as expected, some HSP60 family members were upregulated, as HSP10 and HSP60 proteins form a single functional unit. In contrast, the levels of transcripts from the ClpB/HSP104 and HSP33 genes, and some HSP70 genes, remained unchanged (Figure 5a,b, green), an expression pattern consistent with the function of cognate HSPs present under normal conditions. In addition, the observed expression patterns were similar in both lineages except for HSP60 gene family members, in which transcripts from some genes were unchanged in CL Brener (Figure 5a, green) but not in the Y strain (Figure 5b), and for HSP10 gene family members, in which one gene was downregulated in the Y strain (Figure 5b, orange) but not in CL Brener (Figure 5a). Interestingly, the transcript levels from some HSP gene family members were downregulated up to 4‐fold at 40°C (Figure 5a,b, orange). The transcript level reduction was mostly observed in HSP40, HSP60, HSP70, and HSP85/90 gene members, and the percentage of gene members exhibiting this pattern ranged from less than 20% to more than 60%.

Most members of the largest HSP gene family in trypanosomatids, HSP40 (Folgueira and Requena 2007), showed unchanged transcript levels, whereas the transcripts of approximately one quarter of each of the HSP40 genes were upregulated or downregulated (Figure 5a,b). A similar pattern of upregulated, unchanged, and downregulated transcripts was observed among members of the HSP60 and HSP85/90 gene families. HSP70 gene family members also showed unchanged or downregulated transcripts after heat shock, but no HSP70 gene showed upregulated transcript levels at 40°C, consistent with a previous study (de Carvalho et al. 1990). Unchanged transcript levels from two HSP70 genes (TcCLB.511211.170_1 and TcCLB.511211.160_1) were more abundant by at least 10–20‐fold, and from the mitochondrial HSP70 gene by at least 5‐fold, than those from other family members.

### 3.5 Surface Protein Gene Expression

The *T. cruzi* genome contains large gene families encoding surface proteins involved in host cell infection and immunoevasion (Berná et al. 2018; Herreros‐Cabello et al. 2020). In differential gene expression analysis of epimastigotes from CL Brener (Figure 5c) and Y strain (Figure 5d) subjected to heat shock, the expression patterns also varied among gene family members, although they were similar in both lineages, with some differences. Trans‐sialidases participate in host cell adhesion and infection, complement inactivation, and some possess both trans‐sialidase and neuraminidase activities. Of the 526 gene members detected, approximately 70% were upregulated, 25% unchanged, and 5% downregulated at 40°C. Similar gene expression patterns were observed for GP63 and the dispersed gene family 1, which are associated with parasite–host interactions and extracellular matrix binding, respectively. Of the 172 GP63 gene members detected, approximately 65% were upregulated, 15% unchanged, and 20% downregulated in clone CL Brener, whereas only 50% were upregulated in the Y strain. Of the 174 dispersed gene family 1 members detected, approximately 60% were upregulated, 30% unchanged, and 10% downregulated in clone CL Brener, whereas only approximately 30% were upregulated in the Y strain. In contrast, most mucin gene members, which are involved in host cell adhesion and immunoevasion, remained unchanged at 40°C, especially in clone CL Brener. Of the 328 mucin gene members detected, approximately 30% were upregulated, 60% unchanged, and 10% downregulated. Of the 531 mucin‐associated surface proteins (MASP) gene members, however, approximately 70% were upregulated, 20% unchanged, and 10% downregulated. Finally, most gene members from the retrotransposon hot spot (RHS) family of proteins, with unknown functions, were either unchanged or downregulated at 40°C. Of the 233 RHS protein gene members detected, approximately 5% were upregulated, 45% unchanged, and 50% downregulated.

## 4 Discussion

In this study, we investigated the transcriptome profile of epimastigotes subjected to heat shock. *T. cruzi* strains show different behaviors during heat shock (Perez‐Morales et al. 2012), and thus we analyzed two strains, CL Brener and Y strain. The lack of statistically significant variation in gene expression at 37°C in both CL Brener and Y strain is consistent with previous suggestions that the parasite is prepared for the infection, as metacyclic trypomastigotes, of the mammalian host, and that the presence of HSPs in *T. cruzi* and other trypanosomatids under non‐heat shock conditions may be part of the differentiation process (Rondinelli 1994; Zilberstein and Shapira 1994). Our results suggest that this preparedness extends to the entire transcriptome and possibly to the parasite's proteome. In contrast, significant variation in gene expression was observed in metacyclic trypomastigotes exposed to a mild heat shock (Cruz‐Saavedra et al. 2020). Different cell forms may have particular response patterns, or this difference may result from strain heterogeneity. The transcriptome profile of *Leishmania major* promastigotes incubated at 37°C shows variation in approximately a third of the parasite's gene transcripts (Rastrojo et al. 2019). This difference may be related to the fact that heat shock at 37°C induces differentiation in *Leishmania* (Barak et al. 2005) but not in *T. cruzi* . On the other hand, the observed pattern of differentially expressed genes at 40°C in our results is consistent with the standard response to severe heat shock, characterized by the reorganization of metabolic pathways and cellular processes. It is also consistent with previous work (Carvalho et al. 1987) showing that the typical general inhibition of protein synthesis is seen at 40°C but not at 37°C and is prevented by incubating the parasite in serum.

According to GO and pathway enrichment analyses, pathways mostly related to the degradation of steroids, hemoglobin, glyconate, and phosphonate and sulfur metabolism such as cysteine biosynthesis were upregulated in epimastigotes incubated at 40°C, and those mostly related to amino acid degradation, acetyl‐CoA biosynthesis, glycolysis, gluconeogenesis, pentose‐phosphate pathway, nucleotide metabolism, tricarboxylic acid cycle, fatty acid degradation, and degradation of cholesterol and other steroids were downregulated. These changes probably reflect a reorganization of energy supply at elevated temperatures, as has been described for other eukaryotes (Richter et al. 2010). Transcripts of several enzymes involved in lipid metabolism were also modulated, likely underlying a homeoviscous adaptation of membrane lipids. Similar changes have also been described in the transcriptome of *T. cruzi* metacyclic trypomastigotes subjected to heat shock at 27°C and 28°C, in which transcripts related to the metabolism of lipids and carbohydrates, evasion of oxidative stress, proteolysis, protein phosphorylation, and ribosomal components are modulated (Cruz‐Saavedra et al. 2020). In contrast, *T. cruzi* epimastigotes subjected to nutritional stress show changes in transcripts related to protein quality or content, the switch from glucose to amino acid metabolism, redox metabolism, and surface proteins (Smircich et al. 2023).

Protein homeostasis is maintained during heat shock through the induction of molecular chaperones and proteases, including HSPs, and several cognate HSPs are present at physiological temperatures to help proteins reach their native state (Richter et al. 2010). A similar pattern was observed in epimastigotes, with transcripts of HSP85, HSP70, and HSP60 abundant even at non‐heat‐shock temperatures. In addition, transcripts from HSP110, HSP60, HSP20, and HSP10 were upregulated at 40°C, whereas transcripts from ClpB/HSP104, HSP33, and HSP70 remained unchanged. Although these unchanged transcripts may originate from cognate HSP genes, it is also possible that the corresponding HSP protein levels are increased through translational regulation. In fact, at 40°C, total HSP70 mRNA levels remain largely unchanged (de Carvalho et al. 1990; Requena et al. 1992), but the polysome‐associated fraction increases, indicating preferential translation of these transcripts (de Carvalho et al. 1990). Some HSP gene families, most notably HSP40 and HSP85, have members with clearly distinct patterns of variation in transcript levels, which is particularly evident in the large HSP40 family. Because different HSP40 family members in other eukaryotes are thought to confer substrate specificity to HSP70, it is tempting to speculate that the differential regulation of the parasite's HSP40 genes during heat shock may reflect the requirements of the corresponding substrates.

Interestingly, transcripts from several members of the HSP40, HSP60, HSP70, and HSP85/90 gene families were downregulated at 40°C. The downregulation of HSP genes has been previously described in breast cancer cells and is considered a dysregulation (Zoppino et al. 2018). The authors show that all small HSP genes are downregulated, whereas only some members of the HSP110, HSP70, and HSP40 gene families are downregulated, a pattern similar to that observed in *T. cruzi* epimastigotes. Reduced HSP70 mRNA and protein levels have also been reported in the gastric mucosa following *Helicobacter pylori* infection, and this reduction was reversed upon elimination of the infection (Konturek et al. 2001). The authors interpret the reduced HSP70 gene expression as an adaptation of the mucosa to the pathogen. However, the physiological significance of the reduced expression of several HSP genes in *T. cruzi* epimastigotes during heat shock remains unclear.

In our results, members of several large gene families exhibited distinct expression patterns following heat shock at 40°C. Some members of the trans‐sialidase, GP63, dispersed gene family 1, mucin, and MASP gene families showed increased transcript levels, which is consistent with previously reported increases in the protein abundance of MASP, GP63, and RHS in epimastigotes (Perez‐Morales et al. 2012). These results are to be expected, as protecting the cell is one of the main functions of these proteins. Increased GP63 transcript levels are also observed in metacyclic trypomastigotes subjected to heat shock (Cruz‐Saavedra et al. 2020). However, we also observed reduced or unchanged transcript levels in other members of these gene families. These varying expression patterns among members of the same gene family are difficult to interpret; they may correspond to different functional subcategories, such as cell adhesion and cell protection. However, further studies are needed to clarify this issue.

A limitation of this study is that we did not assess the parasite's proteome during heat shock. Although in general, protein‐level variations roughly follow mRNA‐level variations (Buccitelli and Selbach 2020), translational control may considerably affect the expression levels of some genes. Future studies should focus on the mechanisms of gene regulation underlying the heat shock response of *T. cruzi* .

## 5 Conclusions

In this study, we showed that the transcriptomes of both CL Brener and Y strain epimastigotes remained largely unchanged after heat shock at 37°C, suggesting that the parasite is prepared for infection of the mammalian host. In contrast, CL Brener and Y strain epimastigotes exhibited a typical transcriptional response to severe heat shock at 40°C. In addition, GO and pathway enrichment analyses showed modulation at 40°C of several pathways that likely reflect a reorganization of the parasite's energy supply, as well as a homeoviscous adaptation of membrane lipids. Finally, HSP and surface protein gene family members showed varied patterns of upregulated and downregulated transcripts at 40°C, although the physiological significance of several of these variations remains unclear.

## Funding

This work was supported by CNPq (573.695/2008‐3), CAPES (1274/2011), and FAPERJ (E‐26/010.001242/2016, E‐26/211.613/2021).

## Supporting information


**Figure S1:** Principal component analysis (PCA) of the expression profiles of *T. cruzi* epimastigotes subjected to heat shock. Scatter plots of PCA of RNA‐seq data from CL Brener (a) and Y strain (b) epimastigotes. Blue, green, and red dots correspond to cells at 29°C, 37°C, and 40°C, respectively.

**Table S1:** Number of sequencing reads mapped to coding sequences of the *T. cruzi* genome.

**Table S2:** Genes of CL Brener epimastigotes with fold‐change variation in transcript levels at 37°C and 40°C compared to 29°C.

**Table S3:** Genes of Y strain epimastigotes with fold‐change variation in transcript levels at 37°C and 40°C compared to 29°C.

## Data Availability

Raw data are available in the GEO database under accession number GSE195949.
